# Attitudes of Crohn’s Disease Patients: Infodemiology Case Study and Sentiment Analysis of Facebook and Twitter Posts

**DOI:** 10.2196/publichealth.7004

**Published:** 2017-08-09

**Authors:** Marco Roccetti, Gustavo Marfia, Paola Salomoni, Catia Prandi, Rocco Maurizio Zagari, Faustine Linda Gningaye Kengni, Franco Bazzoli, Marco Montagnani

**Affiliations:** ^1^ Department of Computer Science and Engineering University of Bologna Bologna Italy; ^2^ Department for Life Quality Studies University of Bologna Rimini Italy; ^3^ Madeira Interactive Technologies Institute Funchal Portugal; ^4^ Gastroenterology Unit Department of Medical and Surgical Sciences University of Bologna Bologna Italy

**Keywords:** health information systems, public health informatics, consumer health information, social networking

## Abstract

**Background:**

Data concerning patients originates from a variety of sources on social media.

**Objective:**

The aim of this study was to show how methodologies borrowed from different areas including computer science, econometrics, statistics, data mining, and sociology may be used to analyze Facebook data to investigate the patients’ perspectives on a given medical prescription.

**Methods:**

To shed light on patients’ behavior and concerns, we focused on Crohn’s disease, a chronic inflammatory bowel disease, and the specific therapy with the biological drug Infliximab. To gain information from the basin of big data, we analyzed Facebook posts in the time frame from October 2011 to August 2015. We selected posts from patients affected by Crohn’s disease who were experiencing or had previously been treated with the monoclonal antibody drug Infliximab. The selected posts underwent further characterization and sentiment analysis. Finally, an ethnographic review was carried out by experts from different scientific research fields (eg, computer science vs gastroenterology) and by a software system running a sentiment analysis tool. The patient feeling toward the Infliximab treatment was classified as positive, neutral, or negative, and the results from computer science, gastroenterologist, and software tool were compared using the square weighted Cohen’s kappa coefficient method.

**Results:**

The first automatic selection process returned 56,000 Facebook posts, 261 of which exhibited a patient opinion concerning Infliximab. The ethnographic analysis of these 261 selected posts gave similar results, with an interrater agreement between the computer science and gastroenterology experts amounting to 87.3% (228/261), a substantial agreement according to the square weighted Cohen’s kappa coefficient method (w2K=0.6470). A positive, neutral, and negative feeling was attributed to 36%, 27%, and 37% of posts by the computer science expert and 38%, 30%, and 32% by the gastroenterologist, respectively. Only a slight agreement was found between the experts’ opinion and the software tool.

**Conclusions:**

We show how data posted on Facebook by Crohn’s disease patients are a useful dataset to understand the patient’s perspective on the specific treatment with Infliximab. The genuine, nonmedically influenced patients’ opinion obtained from Facebook pages can be easily reviewed by experts from different research backgrounds, with a substantial agreement on the classification of patients’ sentiment. The described method allows a fast collection of big amounts of data, which can be easily analyzed to gain insight into the patients’ perspective on a specific medical therapy.

## Introduction

Patient opinions are highly valued in many medical studies for the assessment of their well-being. However, it is not always easy to collect patients’ feedbacks for clinical studies. Interestingly, the advent of means of one-to-many communication, including the Web and social media, support peer-to-peer and one-to-many exchanges and comparisons of patients’ experiences and feelings. Such Web-based tools have also radically changed the scenario in front of caregivers; patients are set in front of many more stimuli and sources of information than before (one-third of adult American citizens consider the Web a diagnostic tool), although no guarantee is granted on the quality of the retrieved information [1-3].

Nonetheless, Web-based anonymity may boost frankness and sincerity, as its privacy is often perceived as absolute, also when compared with the direct patient-doctor interactions. Sharing their experiences on the Web, patients provide a very useful knowledge base of insights to both rookies and medical researchers [4]: the former could learn how to handle given situations, and the latter could gather more sincere and unbiased feedback or even acquire further knowledge in their field of clinical study.

Although the reasons for understanding what is shared on the Web in relation to a given disease are clear, no well-established method exists today. Challenges, in fact, may be found and are not limited to (1) data gathering, (2) filtering of any unwanted or unnecessary information, (3) key topics individuation and interpretation, and (4) comparison to any related state-of-the-art in medical research.

An open question amounts to understand what the medical community could learn from the information that is shared on the Web [5-8]. Such new interesting area of research is part of the novel infoveillance and infodemiology fields. A few studies have considered such a problem in relation to different chronic diseases [9-16]. However, to the best of our knowledge, a general approach to this class of problems, based on the use of a combination of different technologies, is missing. This requires expertise that cannot stop to the medical or statistical fields but must also include techniques developed in computer science in addition to others from econometrics, ethnographic research, and psychometrics areas of study.

We borrowed the techniques from the aforementioned scientific areas to investigate a well-defined community of chronic illness patients affected by Crohn’s disease. The choice of such a community is motivated by the following important fact: Crohn’s disease is a chronic illness with increasing incidence, especially in western countries where it is often diagnosed in young people (in the age range of 15-30 years) who typically spend a lot of time on the Web [17]. Crohn’s disease is therefore a good study model for our purposes.

The method that we present builds upon steps that we have previously developed [18,19]. In an initial analysis [18], computer science and econometrics techniques led us to find that (1) Crohn’s disease patients share more frequently information on Facebook pages rather than in Twitter streams, and (2) the pharmaceutical treatment that is most often cited, in both positive and negative terms for Crohn’s disease, is Infliximab. Further contributions have been made [19], where we put our findings in relation with small and large scale medical trials.

Now, the logic and contribution of this paper is to present a method on how Web-based patient information could be obtained and evaluated. To this aim, the following research questions (RQs) are considered:

1. Between Twitter and Facebook, which social media platform do people post on most frequently?
2. Which topics trigger the most patient reactions (eg, medical therapy satisfaction or dissatisfaction)?
3. What kind of attitude do patients have toward the most debated topic (eg, positive, neutral, or negative)?

The results of this study should be integrated with traditional research approaches to help clinicians understand patients’ perspectives.

## Methods

**Figure 1:**
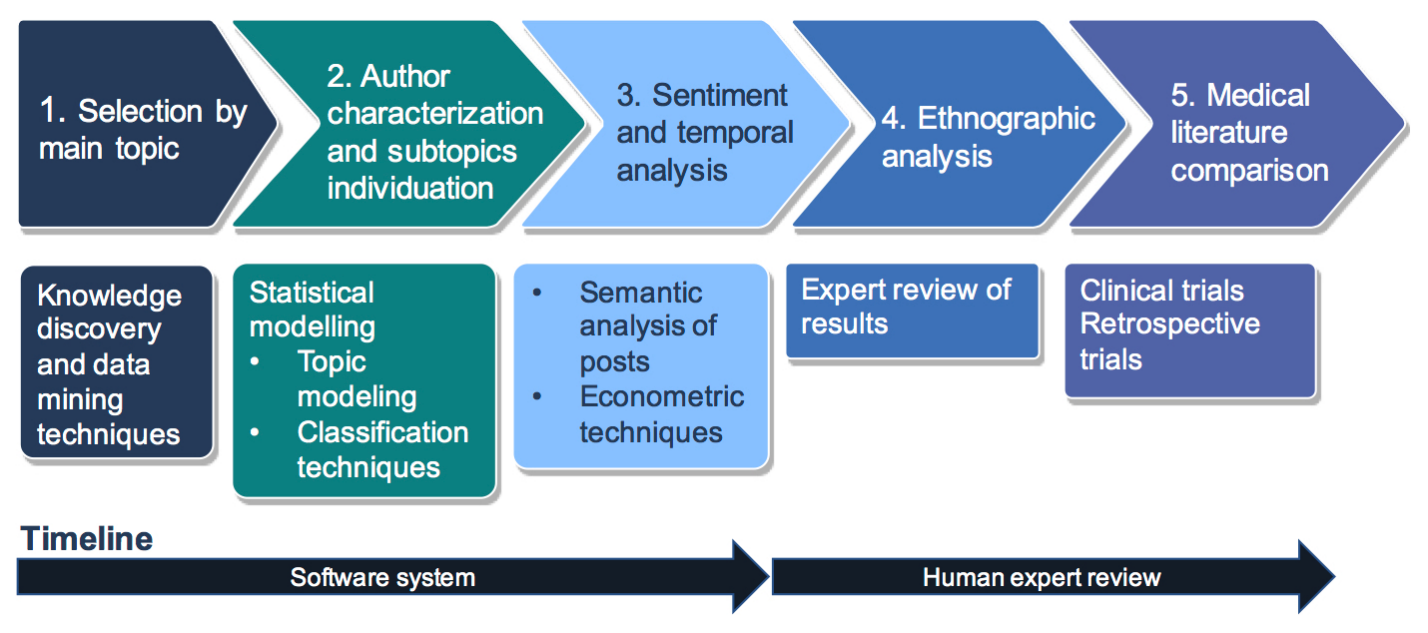
Web-based patient feedback analysis.

Answers to RQ1, RQ2, and RQ3 were obtained following the methods delineated in Figure 1, where the problem of finding and analyzing Web-based data involves two steps. The first one (leftmost part of the timeline) relies completely on software components, whereas the second includes the intervention of human operators. Why this architectural choice has been made will become clear in the following subsections.

### Topic Selection

To understand where patients share their experiences, we implemented a selection procedure (selection by main topic in Figure 1), a well-known operation in data mining and knowledge discovery [20,21]. In fact, no a priori knowledge may be available regarding where patients prefer sharing their experiences. Often the burden of such discovery process is very limited, as many forums and social media pages are often entirely dedicated to the discussion of given diseases. Hence, it is often simple to carry out this step accessing a great quantity of relevant data.

However, often posts are not written by patients (ie, many report scientific news or drug advertisements). Such a problem requires the implementation of mechanisms capable of identifying sites where patients publish their experiences. In our analysis of Crohn’s disease patients, this has been done resorting to two different techniques known for the uncovering of social spammers [22,23].

The first technique simply amounts to identify nonhuman Web-based posts from the number of duplicate ones that may be associated to a single user account. In fact, duplicates are frequently associated to those accounts which are dedicated to post news or advertisements [22]. The second amounts to analyze the behaviors of single writers [23]. To this aim, we performed an additional test, assessing the role of the most prolific users on both social media (please note that this test could be performed automatically by a computer program) to determine whether they were patients or not.

### Subtopic Individuation

The second step of interest is that of shaping the corpus of acquired data, characterizing and modeling it in terms of subtopics of interest. Four different subtopics have been individuated: lifestyle, symptoms, treatments, and side-effects, used to define four corresponding dictionaries. Such approach is consistent with previous works on medical data mining [24-26]. Within the lifestyle subtopic, we included all those terms that are related to the behavior of a patient (eg, food consumption habits and smoker or nonsmoker). Symptoms, treatments, and side-effects contain, instead, the words representing the distinctive signs of a disease (eg, fever and high pressure), the names of the medications utilized to contrast it (eg, tylenol and paracetamol), along with any related side-effect (eg, dizziness), respectively.

For the sake of completeness, we note that the number of subtopics, in general, may be any. The area of topic individuation and modeling is an active area of research whose developments may prove to be very useful in such context, to reveal the topics treated in a corpus of posts [27]. In text data mining, the creation of dictionaries is called feature selection. A wide variety of feature selection methods exist. One of the most common methods for quantifying the discrimination level of a feature is the use of a measure known as the Gini-index [28]. In essence, let *p*_i_(*w*) be the conditional probability that a document belongs to class *i*, given the fact that it contains the word *w.* The Gini-index for word *w*, denoted by *G* (*w*), is defined as *G(w)=∑ p*_i_*(w)*^2^ where *k* amounts to the number of classes. The value of *G* (*w*) always lies in (*1/k, 1*), with higher values of *G* (*w*) associated to a higher discriminative power of the word *w*. Such an approach is very general, however. For the very specific situations, say a situation where we are interested at selecting those posts where users mention a specific medication, setting *w*=medication name results a reliable indicator of an ongoing exchange regarding this topic.

### Sentiment Analysis

After a topic has been identified and posts containing words pertaining to that topic selected, an additional step is performed to determine the relationship of a patient with the given topic. To this aim, sentiment analysis techniques have been exploited, as their performance is progressively becoming more accurate and reliable [29-33]. In this work we used University of Pittsburgh’s OpinionFinder, but additional resources are freely available for the assessment of sentiment values in posts [34]. For example, the Apache OpenNLP framework could be utilized to classify text into predefined categories resorting to the maximum entropy algorithm [35]. Standford’s StanfordNLP, in addition, is a tool trained with 215,154 phrases with fine grained sentiment labeling [36].

Subsequently, in order to verify the correlation between given topics and given sentiment values, econometric approaches (eg, Granger causality) have been employed. Notably, we borrowed such an approach from social media data mining applied to stock exchange analysis [37]. Logistic regression approaches also appear viable for such a domain [38]. Simpler approaches could also be employed to verify the co-occurrence of negative or positive expressions with given key terms. In essence, various statistical analysis methodologies can be utilized to evaluate the importance of a given topic within post sentiment values.

### Ethnographic Analysis

The use of software components in the chart shown in Figure 1 ends with the sentiment analysis step. After individuating the topic of greatest interest for patients, we analyzed, by ethnographic approach, the qualitative feeling of the patients on the specific issue. Since the use of the Infliximab therapy was the most discussed topic (see below), we adopted a 3-valued Likert scale to assess the sentiment value of a patient toward Infliximab [39]. A value of 1 was attributed to positive, 0 to neutral, and −1 to negative feelings. Because we wanted to investigate the reliability of such manual assessment, we compared the ethnographic analysis performed by a computer science researcher and a senior gastroenterologist. We then analyzed the concordance of such assessments using the square weighted Cohen’s kappa coefficient method. Additionally, we also assessed the patients’ feelings according to the 3-point Likert scale using our software system, which relied on OpinionFinder.

## Results

### Topics, Subtopics, and Sentiment Analysis

In 2014, 71% and 23% of adults on the Web used Facebook and Twitter, respectively [40]. Because of this fact, our attention focused on the posts that could be found on these two social networks. In fact, such two social networks have the potential of providing spontaneous and uncontrolled patients’ opinions differently from thematic and moderated Web-based platforms specifically designed for patients.

To begin our analysis (RQ1), we searched for the “crohn” keyword to select relevant tweets on Twitter and to individuate Crohn’s Facebook public pages from their title. By these means, we found over 26,000 tweets and almost 56,000 posts on Facebook published from October 2011 to August 2015. A further analysis of such posts let us conclude that the feedback of real patients is more easily found on Facebook rather than on Twitter (such result corroborates similar findings) [18].

Concentrating on Facebook, we found the terms that belonged to the four subtopics of interest, and we selected those that appeared at least 50 times (Table 1). Such dictionaries include both specific terms (eg, diarrhea or abdomen) but also generic ones that are related to the subtopic (eg, suffer or symptom). Please note that our results are consistent with the findings obtained using a different methodology based on metadata analysis from PubMed [24].

The analysis of such subtopics produced three terms (RQ2), namely Adalimumab, Azathioprine, and Infliximab, which triggered the longest and most vibrant discussions among people. We then adopted Granger and sentiment analysis to investigate which one of these three terms was more strictly related to the patients’ feelings. Infliximab was the most sentiment-related term, with a statistical significance association to either positive or negative feelings (*P*=.04 and *P*=.01, for positive and negative feeling, respectively).

### Ethnographic Analysis of Posts Related to Infliximab

Inspired by ethnographic approaches [41], we performed an expert review of the threads of 261 posts containing the keyword Infliximab (such posts are available in the study by Roccetti M. et al [42]). Two different groups of experts read all the posts containing the term Infliximab (or alternative trade names such as Remicade) to either confirm or deny the positive or negative evaluations assigned to those posts by the employed software system. 

**Figure 2:**
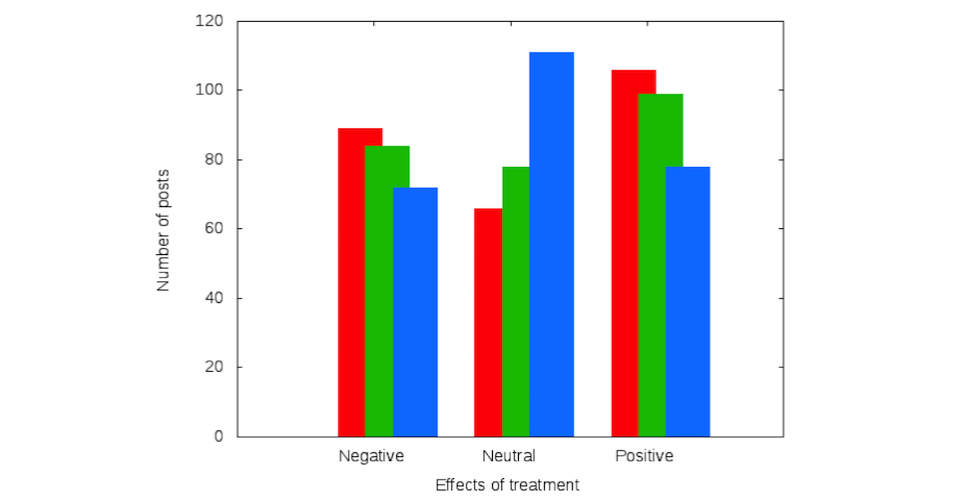
Computer scientist (red bars), senior gastroenterologist (green bars), and software classification (blue bars).

The classification performed by both groups (computer scientist and senior gastroenterologist) confirmed that a relevant fraction of patients treated with Infliximab were not fully satisfied. The outcome (RQ3) is portrayed in Figure 2.

**Table 1:** Subtopic dictionaries.

Subtopics	Dictionary
Lifestyle	Alcohol, bacteria, butter, cake, cell, chocolate, coffee, drink, eggs, food, gene, honey, lactose, map, meat, milk, pasta, smoke, sugar, tnf, virus, vitamin, and wine.
Symptoms and body parts	Abdomen, abscess, agony, anal, anxiety, appetite, arthritis, attack, belly, bladder, bleed, blood, bone, bowel, butt, colitis, constipation, cramp, damage, deficiency, depression, diabetes, diarrhea, digestion, disorder, exhausted, fever, fistula, flare, flu, gastro, grow, hurt, infection, inflamed, intestine, liver, mouth, muscle, nausea, pain, psoriasis, rectum, scar, severe, sleep, stress, suffer, symptom, tired, toilet, ulcer, and vomit.
Treatments	Adalimumab, aloe, antibiotic, asacol, azathioprine, budesonide, calcium, cannabis, capsule, certolizumab, cimzia, colectomy, colonoscopy, colostomy, diagnosis, diet, doctor, dose, drain, entocort, enzyme, fda, ferment, ginger, gp, health care, hospital, humira, ileostomy, imuran, infliximab, infusion, injection, kefir, marijuana, medication, medicine, mercaptopurine, methotrexate, morphine, mri, natural, nutrition, operation, oral, organic, paleo, pentasa, powder, prednisolone, prescribed, prescription, probiotic, rafton, remedy, remicade, resection, reversal, scd, solution, specialist, steroid, surgeon, surgery, test, therapy, transplant, treat, and visit.
Side-effects	Complications, effect, lupus, reaction allergy, and skin.

**Table 2:** Square weighted Cohen’s kappa coefficient (w2K) for the interrater agreement. The number of patients corresponding to the attributed score (−1, 0, 1) is indicated for each different observer (senior gastroenterologist vs computer science expert). The interobserver agreement was substantial: 87.36% (w2K: 0.6470).

	Computer science expert score			
Senior gastroenterologist score	−1	0	1	Total
−1	62	17	5	84
0	22	40	16	78
1	11	13	75	99
Total	95	70	96	261

Both expert reviews point to the same conclusions, as confirmed by interrater agreement statistical analysis (data reported in Table 2). The interrater agreement was performed using a square weighted Cohen’s kappa coefficient (w2K). A substantial agreement (w2K=0.6470, corresponding to 87.36%) was found comparing the computer scientist versus the senior gastroenterologist evaluation of patients’ global sentiment. This result indicates that the evaluation of the feeling that was communicated by a post was independent of the scientific background of the reader, although the senior gastroenterologist tended to classify as neutral a slightly larger share of posts, as not deemed relevant from a clinical point of view.

The classification performed by our software system, instead, provides a different outcome than those given by the computer science expert and by the senior gastroenterologist. In fact, the number of posts classified as neutral increase, as the sentiment analysis algorithm was evidently unable to determine with a precision similar to a human being the underlying meaning of a piece of text. Nonetheless, the proportion between positive and negative posts remains comparable, showing that the algorithmic tool could be useful to determine the existence of situations where positive and negative remarks concerning Infliximab were made.

## Discussion

The availability of big data from social networks may be seen as an important source of information in medical research, alternative to the traditional sources of information [43,44]. Obviously, there are limitations, as patient characteristics (eg, age and sex) are often unknown.

We used social networks to analyze the perception of therapies by Crohn’s disease patients. Crohn’s disease has been chosen because of its well-defined features of chronic and sometimes disabling disease, with a strong impact on the quality of life of patients. Additionally, Crohn’s disease is typically diagnosed in young patients (in the age range of 15-30 years), an age group of frequent social network users.

This work expands our previous studies, to propose a method to analyze the information posted on the Web. An important point of this work is that we use data derived from external observation of patients’ spontaneous opinions during their daily lives. From this perspective, this study is a meticulous observation of the big data that a social network like Facebook may supply.

Our previous analyses revealed that Facebook (RQ1), with respect to Twitter, is the social network in which it is easier to find Crohn’s disease information [18]. Our further studies individuated Infliximab as the most debated drug (RQ2), with both positive and negative sentiments among Crohn’s disease patients [19]. This result was justifiable considering that Infliximab has been the first biological treatment (ie, monoclonal antibody) capable of strongly improving Crohn’s disease management, with a rapid diffusion in the clinical setting. In addition, social networks usage started a few years after the 1998 approval of the Infliximab therapy for Crohn’s disease patients, and this chronological coincidence possibly boosted the discussion on sites such as Facebook. Notably, a good match was found between the sentiment assessments in relation to Infliximab obtained, with the ethnographic analyses performed by either computer science or gastroenterology experts (RQ3). This indicates that a data mining approach provided material of simple interpretation, regardless of the analysts’ scientific and professional background. This represents a good starting point to provide a completely automated approach for the analysis of such data, in substitution of the final ethnographic step performed in this work. Another important finding is that our ethnographic results are in substantial agreement with the medical literature. In fact, medical trials involving large numbers of patients (large-scale retrospective trials) exhibit a percentage of those who experienced a negative reaction to Infliximab falling between 20-40% [45,46].
